# Quit in General Practice: a cluster randomised trial of enhanced in-practice support for smoking cessation

**DOI:** 10.1186/1471-2296-11-59

**Published:** 2010-08-12

**Authors:** Nicholas Zwar, Robyn Richmond, Elizabeth Halcomb, John Furler, Julie Smith, Oshana Hermiz, Irene Blackberry, Ron Borland

**Affiliations:** 1 School of Public Health and Community Medicine, University of New South Wales, Sydney NSW 2052 Australia; 2 School of Nursing and Midwifery, University of Western Sydney, Locked Bag 1797, Penrith South DC Sydney NSW 1797, Australia; 3 Primary Care Research Unit, Department of General Practice, University of Melbourne, 200 Berkeley St, Carlton, Melbourne Victoria 3053, Australia; 4 Australian Centre for Economic Research on Health, Australian National University Canberra, ACT 0200, Australia; 5 Centre for Primary Health Care and Equity, University of New South Wales, Sydney NSW 2052, Australia; 6 Cancer Council Victoria 1 Rathdowne St, Carlton, Melbourne Vic, 3053, Australia

## Abstract

**Background:**

This study will test the uptake and effectiveness of a flexible package of smoking cessation support provided primarily by the practice nurse (PN) and tailored to meet the needs of a diversity of patients.

**Methods/Design:**

This study is a cluster randomised trial, with practices allocated to one of three groups 1) Quit with Practice Nurse 2) Quitline referral 3) GP usual care. PNs from practices randomised to the intervention group will receive a training course in smoking cessation followed by access to mentoring. GPs from practices randomised to the Quitline referral group will receive information about the study and the process of written referral and GPs in the usual care group will receive information about the study. Eligible patients are those aged 18 and over presenting to their GP who are daily or weekly smokers and who are able to give informed consent. Patients on low incomes in all three groups will be able to access free nicotine patches.

Primary outcomes are sustained abstinence and point prevalence abstinence at the three month and 12 month follow-up points; and incremental cost effectiveness ratios at 12 months. Process evaluation on the reach and acceptability of the intervention approached will be collected through Computer Assisted Telephone Interviews (CATI) with patients and semi-structured interviews with PNs and GPs.

The primary analysis will be by intention to treat. Cessation outcomes will be compared between the three arms at three months and 12 month follow-up using multiple logistic regression. The incremental cost effectiveness ratios will be estimated for the 12 month quit rate for the intervention groups compared to usual care and to each other. Analysis of qualitative data on process outcomes will be based on thematic analysis.

**Discussion:**

High quality evidence on effectiveness of practice nurse interventions is needed to inform health policy on development of practice nurse roles. If effective, flexible support from the PN in partnership with the GP and the Quitline could become the preferred model for providing smoking cessation advice in Australian general practice.

**Trial Registration:**

ACTRN12609001040257

## Background

Tobacco remains the most common preventable cause of death in the world today. The World Health Organization estimates that tobacco killed 5.4 million people globally in 2008 and on current trend this will rise to 8 million deaths per year by 2030 with more than 80% of deaths occurring in the developing world [1]. Despite reduction in prevalence of smoking in the Australian population, tobacco use and dependence remains the risk factors associated with the greatest disease burden, accounting for 9.5% of the total burden in men and 6% in women [2]. More than one in six Australians continue to smoke each day, and tobacco smoking is responsible for the premature deaths of about 16,000 Australians each year [3]. Less than 10% of Australian smokers consistently deny any interest in quitting and approximately 40% report attempting to quit in the previous year [4]. Unfortunately unaided quit attempts have a low success rate of only 3-5% [5].

### Suitability of general practice for a quit smoking intervention

General practice is well suited for supporting smoking cessation. Around 85% of the Australian population visits a general practitioner (GP) at least once a year [6]. There is variation in reported rates of smokers presenting in general practice, with between 19.9% -35% of adult patients reported as currently smoking [7,8]. The familiar environment of general practice and the sustained relationships between patients and providers can provide an environment conducive to effective promotion of behavioural risk factor modification [9].

### Current general practice intervention

There is clear evidence that smoking cessation advice from a physician has an effect. The Cochrane review of 17 trials of brief advice versus no advice estimates that brief advice increases absolute rates of cessation at one year follow-up by about 2.5% compared to usual care [10]. This effect can be increased substantially if brief advice is combined with other evidence based support such as pharmacotherapy [11].

GPs in Australia as elsewhere have been encouraged by clinical practice guidelines [11] to offer smoking cessation advice and support, and some have attended training [12]. Despite this the number of patients who report receiving advice on smoking cessation from GPs is low [13]. In an Australian study of GPs' use of evidence-based approaches only 32% provided written materials and 28% set a 'quit date' [14]. Barriers raised by GPs to engaging in greater efforts in smoking cessation include: perception of lack of effect; lack of GP time; lack of GP skills; reluctance to raise the issue due to perceived patient sensitivity about smoking; and perceived lack of patient motivation [15].

### Quitline referral

An alternative to in-practice support from the GP is for GPs to identify smokers, provide brief advice and actively refer interested patients to the telephone Quitline. A study in Australia which compared standard in-practice management to this active referral to Quitline has shown that at 3 month follow up patients randomised to the referral intervention had a higher rate of sustained abstinence (12.3% compared with 6.9%). At 12 month follow-up patients in the referral intervention had a higher rate of sustained abstinence (6.5% compared to 2.6%), however this did not reach statistical significance [16]. The researchers concluded that GPs referring smokers to an evidence based telephone cessation service can result in increased cessation.

### Practice nurses and cessation support

An innovative model for enhanced smoking cessation support is provision of advice in the practice by general practice nurses. Practice nursing is rapidly emerging in Australia and is making a considerable contribution to capacity in primary care [17].

In Australia the practice nurse workforce has grown rapidly since 2003. Almost 60% of Australian general practices now employing a practice nurse [18] and practice nurses are increasingly regarded as core members of general practice teams [19]. The Australian Government has committed over $A230 million to support practices to employ practice nurses, however, their potential impact has been constrained by poor role descriptions, inadequate funding models, negative GP attitudes and unclear legal implications of the nursing role [20].

Although consumers support the practice nurse role in principle, there is no research to date on consumer acceptability of specific practice nurse interventions [21]. There is also a need for trials to test the impact of practice nurse interventions to ensure that the significant investment being made in expanding the practice nurse workforce achieves maximum impact on improving quality of care and patient outcomes [22]. Face-to-face support for smoking cessation provided within the practice primarily by a trained practice nurse may appeal to different groups of smokers who are less likely to use a telephone service such as people from culturally and linguistically diverse backgrounds (CALD). Support in the practice has the potential to be even more effective than referral to an outside service, given the familiar environment and setting of the practice within the local community [9].

Several studies have explored the effectiveness of involving the practice nurse in supporting smokers to quit [23-31]. In a study by Vetter et al there was a significant benefit at six month follow up [26]; another study demonstrated a significant reduction in smoking status [29] and a third study showed a reduction in the number of cigarettes smoked per day [28]. All other studies showed no significant difference. Limitations of these studies included the low uptake of the nursing intervention [23], research designs that provided only a one off nurse consultation and a lack of follow-up [24]. Additionally, there were low retention rates amongst smokers in these studies. The difficulties in retention of this group as study participants may have been responsible for the small effect sizes and lack of significance seen in some studies [32].

### Cost effectiveness of smoking cessation interventions

Smoking cessation strategies have been shown to be highly cost effective compared to many pharmacological, surgical and hospital treatments or services [33]. A recent systematic review of studies in a range of settings adjusted for variation in program effects and costing methods found cost effectiveness ratios ranged from US$490 to $US15280 per life-years saved (LYS) in different settings [34].

Face to face interventions are the most common strategies. A recent review of cost effectiveness studies for such face to face health behaviour interventions addressing smoking cessation found favourable cost effectiveness ratios for smoking cessation programs compared to preventative pharmaceutical and invasive interventions [35].

More intensive interventions may be more effective but also more costly [10]. Many studies omit disadvantaged populations which are known to have higher levels of smoking [10]. Health gains are likely to be larger in such groups. Hence it is proposed that although more intensive interventions may be more costly, cost effectiveness will still be very favourable as a result of the high health gains. The cost effectiveness analysis in this study will compare the more intensive interventions to usual care.

### Aims and objectives

This study will test the uptake and effectiveness of enhanced in-practice support for smoking cessation. The in-practice intervention (**Quit with Practice Nurse**) involves flexible support for quitting provided primarily by the practice nurse in partnership with the patient's GP and the Quitline. The number and diversity of smokers receiving intensive intervention will be optimised by offering a flexible package of support from the nurse, GP and Quitline to match the patient's needs. The rationale is that delivering more integrated and intensive quitting support will be more effective and offering flexibility in service provision will reach more smokers from a wider range of demographic, socioeconomic and cultural backgrounds.

The impact of the 'Quit with Practice Nurse' intervention will be compared contrasted with an alternative intervention group as well as with a control group. The alternative intervention is referral to an evidence based telephone support service (Quitline Referral Intervention). The control group is usual care by the GP (control group). Referral to a specialized cessation service such as the Quitline represents the major alternative approach to supporting cessation. An important strength of this study is the comparison of the new approach to both a referral model and usual care.

### Specifically the study will

• Evaluate uptake of the 'Quit with Practice Nurse' intervention versus the referral intervention and the control group in terms of the number of patients, demographics, socio-economic status, language and ethnicity, smoking history and level of nicotine dependence. *Hypothesis: the 'Quit with Practice Nurse' intervention will reach more smokers because it will meet the needs of a greater range of smokers with different demographic, socioeconomic and cultural characteristics than referral model or standard in-practice GP management*.

• Compare the effect on cessation of 'Quit with Practice Nurse' versus 'Quitline Referral' and the control group. *Hypothesis: the 'Quit with Practice Nurse' model will achieve higher quit rates as more patients will receive a cessation intervention that meets their individual needs*.

• Examine the cost effectiveness of the three approaches and their components from the perspective of the health care sector. *Hypothesis: the more intensive intervention may be more costly than usual care or 'Quitline Referral' but any higher costs of the 'Quit with Practice Nurse' intervention will be justified by its higher uptake and quit rate*.

• Assess the acceptability to patients of the 'Quit with Practice Nurse' intervention. *Hypothesis: the 'Quit with Practice Nurse' intervention will be acceptable to patients as it offers a flexible package of support to meet patient needs*.

• Assess acceptability and sustainability of practice nurse assisted support to quit from the perspective of practice nurses and GPs. *Hypothesis: the 'Quit with Practice Nurse' intervention will be acceptable to practice nurses and GPs as it provides an important new role for practice nurses and provides GPs the option of referral within their practice*.

## Methods/Design

This study is a three arm cluster randomised trial involving general practices with practice nurses located in Sydney and Melbourne, which are Australia's two largest cities. Recruitment will expand if necessary to other parts of New South Wales and Victoria. Practices will be allocated to one of three groups 1) Quit with Practice Nurse 2) Quitline Referral 3) usual care control group.

### Recruitment

#### General practice recruitment

Practices which employ at least one practice nurse will be eligible to participate. Eligible practices will be identified with the assistance of local general practice networks (local GP organisations). All GPs working in these practices will be approached by mailing an initial invitation letter followed by a telephone call from one of the chief investigators. GPs expressing interest on the phone will be visited by project staff to explain the project to GPs and practice nurses and to gain informed consent.

Randomisation to intervention groups will follow procedures outlined in the CONSORT statement [36] and will be performed by a researcher independent of the project team. Randomisation will occur in permuted blocks of size three using a system of sealed envelopes. Following randomisation, practices are notified of their allocation prior to the commencement of patient recruitment. Practices in the control group are given copies of smoking cessation guidelines [11]. Practices in the active referral arm are in addition given copies of active referral sheets with which to make referrals to the Quitline. Practices in the 'Quit with Practice Nurse' group are scheduled for the practice nurse to attend the one day training course (see below).

#### Patient recruitment

Eligible patients are those aged 18 and over presenting to their GPs who are daily or weekly smokers. Potential participants will be excluded if they meet any of the following criteria: unable to give informed consent (poor physical and/or cognitive state), insufficient command of English to comprehend the consent process and/or data collection questions. A research assistant will be attached to each participating practice for a two week recruitment period and approach patients in the waiting room prior to the consultation with the GP. Initially the research assistant will assess patient eligibility and, if eligible, patients will be given a copy of participant information statement. Patients providing a written consent will be asked to complete the baseline data questionnaire at this time. Patients take notice of their participation into the GP consultation. GPs respond to this notification depending on their group allocation.

This method of recruitment maintains a separation of baseline data collection from the intervention. Waiting room recruitment has previously been used successfully by members of the research team in studies on smokers, [37,38] risky drinkers [39] and overweight and obese patients [40].

### Intervention

#### Quit with Practice Nurse

This intervention involves the practice nurse, GP and Quitline working in partnership with the patient to provide flexible assistance that meets the needs of individual smokers. The GP identifies smokers and their willingness to quit and offers brief advice. Patients with any interest in quitting are referred to the practice nurse. The practice nurses will see the patient for an initial assessment visit. At this assessment the practice nurse gathers information about patient demographics, smoking behaviour, stage of readiness to quit, previous quit attempts, nicotine dependence (using Fagerstrom test), previous use of pharmacotherapy and perceived barriers to quitting. Patients are assisted to develop a quit plan and (in consultation with the GP) encouraged to use pharmacotherapy according to recommendations in clinical practice guidelines [11,41]. Patients are then offered a flexible package of ongoing support. Those able to attend for face-to-face counseling are offered a series of weekly (three follow-up visits) visits with the practice nurse. At these visits patients are provided behavioural counseling, support in use of pharmacotherapy, relapse prevention advice and encouragement of social support as shown in table 1.

**Table 1:** Practice Nurse Visits

	Visit 1	Visit 2	Visit 3	Visit 4
Smoking assessment	✓			

Nicotine dependence assessment	✓			

Pharmacotherapy discussed	✓	✓	✓	✓

Nicotine patches offered to eligible patients	✓		✓	✓

Quit support plan developed	✓			

Cessation counseling support	✓	✓	✓	✓

Patients in this group who are unable to attend for face-to-face consultations or who prefer telephone counseling to support their quit attempts will be referred to the Quitline using a faxed referral system. As in the referral intervention described below, patients are contacted and offered services to meet their needs. Patients are encouraged to use the proactive callback service which has been shown to be more effective than reactive counseling [42]. Feedback is provided by the Quitline to the practice on acceptance, use and outcomes of services offered to facilitate the ongoing management of the patient in the practice. Patients who are able to attend one or two practice nurse visits will be offered a combination of nurse counseling and referral to Quitline.

#### Nurse training for 'Quit with Practice Nurse' intervention

Training for the practice nurses will consist of a one day training workshop where the nurses are educated in: 5As approach to smoking cessation counseling (Ask, Assess, Advise, Assist, Arrange follow-up); basics of motivational interviewing; nicotine dependence; smoking cessation pharmacotherapy and resources for smoking cessation including Quitline services. The practice nurses will receive mentoring over the course of the project from an experienced Quitline counselor who they can contact for advice. The counselors involved will attend the practice nurse training sessions to establish this mentoring relationship

#### Active referral to Quitline

The '**Quitline Referral' **intervention involves the GPs identifying smokers and their willingness to quit and offering brief advice. Patients with any interest in quitting are offered referral to the Quitline. Patients who consent will have their referral faxed to the Quitline and are provided with a brochure on Quitline services. Patients are telephoned by the Quitline and offered services to meet their needs. Patients who express interest in quitting are offered a series of proactive callback counseling/advice sessions [42]. Referring practices are provided with feedback from the Quitline on acceptance, use and outcomes of services offered to facilitate ongoing management of the patient. GPs are expected to provide advice on use of medications and prescriptions where appropriate.

#### Control Group

The control group involves GPs identifying smokers and their willingness to quit and offering assistance in accordance with their usual practice. This should include advice on use of medications to quit and prescriptions where appropriate. It may involve advice provided by themselves within the practice or referral to the Quitline or both where the GP deems it appropriate, but no provision is made to facilitate either. Based on previous work, levels of either referral or intense counseling within practice interventions are likely to be very low [16].

#### Smoking cessation pharmacotherapy

Patients in all three groups will be encouraged to use smoking cessation pharmacotherapy based on best practice guidelines [11,41]. For patients in all three groups who are on low incomes and therefore eligible for subsidised medicines under the Australian Government Pharmaceutical Benefit Scheme (PBS) the project will fund access to nicotine patches. This targeted subsidy will not disrupt the ecological validity of the study as a test of the different modes of cessation support as it will be offered equally across all three arms of the study

#### Outcome measures

Patient level measures will be assessed at baseline, three months and 12 months (the schedule of data collection is shown in table 2. At baseline patients will be asked to complete a paper questionnaire assessing demographics including age, sex, language spoken at home, ethnicity, education levels, occupation, current level of smoking, nicotine dependence, quitting history and presence of co-morbidities. Three month and 12 month data will be collected by Computer Assisted Telephone Interview (CATI) by trained interviewers who are blind to intervention group until after the outcome data is collected.

**Table 2:** Schedule of data collection

	At recruitment	3-months	12-months
Age, gender, language spoken at home,	X		
Smoking history	X	X	X
Nicotine dependence	X	X	X
Sustained abstinence		X	X
Point prevalence abstinence		X	X
Quit attempts			X
Intervention costs		X	X
Process evaluation data from patients			
Process evaluation data from PN, GPs and Quitline counselors			X

#### Primary outcomes

Uptake of the intervention in each group and characteristics of patients - age, sex, language spoken at home, ethnicity, education levels, occupation, smoking history, level of smoking and nicotine dependence.

Smoking cessation rates: sustained abstinence defined as patients reporting abstinence of ≥ one month at the three month follow-up and ≥ 10 months at the 12 month follow-up; and point prevalence abstinence defined as ≥ seven days of sustained abstinence at the three month and 12 month follow-up points. Validation of smoking cessation will not be undertaken as reviews confirm the accuracy of self-report measures, and conclude that validation is unnecessary in trials where there is no strong association between the interviewer and respondent [43]. Number and duration of quit attempts will be a further outcome measure as it is known that the key effect of advice from a health professional is to encourage the smoker to try to quit [10,11].

Health economic outcomes: the economic evaluation will compare the flexible support intervention 'Quit with Practice Nurse' and the 'Quitline referral intervention' with standard 'in practice management by the GP' (control group.) Cost analysis will be from the perspective of the health care sector and include direct costs of the intervention, including for recruitment, and for the intervention (GP costs, practice nurse costs, nurse training costs, telephone counseling costs, pharmaceutical costs, and overheads such as room rents).

#### Process outcomes

Quantitative and qualitative data on process outcomes will be collected as part of the CATI of participating patients in each of the three arms at the three month follow-up point. These questions will be asked only after the primary outcome data on abstinence has been collected. In the CATI patients in all three arms will be asked about uptake including barriers and enablers to uptake, extent and nature of the intervention received including use of smoking cessation pharmacotherapy (type of pharmacotherapy used and duration of use), acceptability and perceived value of the smoking cessation support received. The interviews will also explore patient perception on the influence of culture, language and socioeconomic status on the acceptability of the intervention approaches. Patients in the 'Quit with Practice Nurse' arm will be asked further questions about the perceived value of the flexible approach to cessation support. These will include their level of awareness or lack of awareness of the flexibility, its importance or otherwise for them, the important factors in the choices made about support options, and their perception on the roles of the providers involved (practice nurse, GP and Quitline counselor).

The acceptability and sustainability of the 'Quit with Practice Nurse' intervention will be evaluated with semi-structured interviews with participating practice nurses, GPs and Quitline counselors.

#### Data Analysis

Analysis for the primary outcomes of sustained abstinence and point prevalence abstinence will be on an intention to treat basis with cases retained regardless of whether they accept or receive the intended intervention. A series of planned imputation strategies for missing data will be used in examining outcomes for missing data. These are 1) analysis will be done on all participating patients, where all patients with missing outcome data will be assumed to be smokers; 2) the last known value carried forward to replace the missing value; 3) an analysis of outcomes for patients with complete outcome data. We will compare cessation outcomes between the three arms at three months and 12 month follow-up using multiple logistic regression. This approach will allow us to adjust for clustering and to assess the effect of potential confounders such as age, sex, socioeconomic status, ethnicity, language spoken at home and nicotine dependence on cessation outcomes.

#### Health economic analysis

The study will estimate incremental cost effectiveness ratios (ICER) of cost per quitter for brief advice and referral to Quitline, and with the flexible support intervention, compared to standard GP care. The ICER will be estimated for the 12 month quit rate for the intervention, less an unaided/natural quit rate. Adjustments to effect sizes will also be made for long term relapse rate among the 12 month quitters.

#### Analysis of qualitative data on process outcomes

Analysis will be based on thematic analysis. Our aim is to identify common themes and issues about barriers and facilitators to the uptake of the intervention from the perspective of both patients and providers. This will allow a richer understanding of the experiences of participants in the study and maximise our capacity to provide meaningful answers to our research questions [44].

#### Sample size

The observed 12 month sustained abstinence outcomes of GP usual care and Quitline referral in Borland et al.'s study in Victoria were 2.6% and 6.5% respectively [16]. The sustained abstinence at six months in a pilot study of practice nurse support was 16%. Assuming this drops to 12% at 12 months we have calculated power to detect a 5.5% difference in sustained abstinence quit rates between 'Quit with Practice Nurse' intervention versus the referral intervention and a 9.4% difference between 'Quit with Practice Nurse' intervention and the usual care control group at one year follow up with 80% power at the 5% significance level. Before adjustment for clustering this requires 471 patients per group to detect the 5.5% difference and 140 per group to detect the 9.4% difference (calculations using STATA software). To adjust for clustering the intracluster correlation coefficient observed by Lennox et al. in a smoking cessation trial in general practice has been used. The intracluster correlation coefficient observed in this study was 0.013 [45]. The resulting design effect = [1+ (size of cluster-1) × intracluster correlation]. In this study we plan to recruit 25 patients per practice so the design effect is 1.31 so the number per arm is 471 × 1.31 = 617 giving a total sample size of 1851. To enroll these participants we will recruit 90 general practices and 2250 patients across New South Wales and Victorian study locations (to allow for approximately 10% drop out rate of practices) and randomise 30 practices to each arm of the study.

#### Ethics Approval

The study has received ethics approval from the University of New South Wales, University of Melbourne and University of Western Sydney Human Research Ethics Committees.

#### Trial Registration

Australian New Zealand Clinical Trials Registry (ANZCTR). Number: ACTRN12609001040257.

## Discussion

This project will test an approach to supporting smoking cessation in general practice based on partnership between the practice nurse, GP and patient to support quitting. This is a new approach in Australia which has not had a system to provide face to face quitting support from specifically trained health professionals in a way that is accessible for most of the population. If successful it would have major benefits for addressing smoking which remains Australia's most important cause of preventable death and disease. The project is highly relevant with current policy direction in developing practice nurse roles and expanding access to Medicare rebates for services provided by practice nurses. Trials of actual interventions involving practice nurses have been identified as an important step in advancing the practice nurse role [22] and this study will provide key evidence to inform this development.

## Abbreviations

GP: General Practitioner; CA: TIComputer Assisted Telephone Interviews; CALD: Culturally and Linguistically Diverse; LYS: Life Years Saved; CONSORT: CONsolidated Standards Of Reporting Trials; ICER: Incremental Cost Effectiveness Ratios; STATA: Portmanteau of the words "statistics" and "data"

## Competing interests

The authors declare that they have no competing interests.

## Authors' contributions

NZ - leading development of the study conceptualisation, design, refining of protocol and write up for publication, RR - input into development of the study conceptualisation, design contribution to protocol publication. EH - input into development of the study conceptualisation with a focus on the role of the practice nurse, design, contribution to protocol publication. JF- input into development of the study conceptualisation, design including qualitative assessment of the intervention, and contribution to protocol publication. JS - development of the study conceptualisation, design with focus on health economic evaluation, contribution to protocol publication. OH - refining of protocol, outcome assessment tools and contribution to protocol publication, IB - refining of protocol, outcome assessment tools, and contribution to protocol publication. RB - input into development of the study conceptualisation, design and contribution to protocol publication. All authors have read and accepted the final manuscript.

## Pre-publication history

The pre-publication history for this paper can be accessed here:

http://www.biomedcentral.com/1471-2296/11/59/prepub
